# Autoluminescent Plants

**DOI:** 10.1371/journal.pone.0015461

**Published:** 2010-11-12

**Authors:** Alexander Krichevsky, Benjamin Meyers, Alexander Vainstein, Pal Maliga, Vitaly Citovsky

**Affiliations:** 1 Department of Biochemistry and Cell Biology, State University of New York, Stony Brook, New York, United States of America; 2 The Robert H. Smith Institute of Plant Sciences and Genetics in Agriculture, Faculty of Agricultural, Food and Environmental Quality Sciences, The Hebrew University of Jerusalem, Rehovot, Israel; 3 Waksman Institute, Rutgers, The State University of New Jersey, Piscataway, New Jersey, United States of America; Indiana University School of Medicine, United States of America

## Abstract

Prospects of obtaining plants glowing in the dark have captivated the imagination of scientists and layman alike. While light emission has been developed into a useful marker of gene expression, bioluminescence in plants remained dependent on externally supplied substrate. Evolutionary conservation of the prokaryotic gene expression machinery enabled expression of the six genes of the *lux* operon in chloroplasts yielding plants that are capable of autonomous light emission. This work demonstrates that complex metabolic pathways of prokaryotes can be reconstructed and function in plant chloroplasts and that transplastomic plants can emit light that is visible by naked eye.

## Introduction

Throughout the evolution, bioluminescence has evolved many times and some thirty independent biological light emission systems are still extant [1], in addition to those that may have not survived evolutionary bottlenecks. Among the diverse light-emitting species are bacteria, dinoflagellates, fungi, and insects. The luciferase enzymes, catalyzing the light-emitting reactions in different organisms, show no homology to each other and their substrates, termed luciferins, are unrelated to each other chemically. Luminous bacteria [2], [3] - all gram negative, motile rods - are the most abundant and widely spread of all light-emitting organisms. They are found as free-living species in the ocean, as saprophytes growing on dead marine organisms, as light organ symbionts in fish and squid and other ecological niches. Almost all luminous bacteria, a majority of them being marine species, are classified into three genera: *Vibrio, Photobacterium*, and *Xenorhabdus* [3], [4]. Bacterial light-emission enzymatic system, encoded by the *lux* operon, is highly conserved among various species of luminous bacteria, with the most common architecture of the *lux* operon represented by *luxCDABEG* [2], [3]. The bacterial luciferase utilizes flavin mononucleotide and a long-chain aldehyde, derived from fatty lipid biosynthesis pathway, as substrates for the light emission reaction. The *luxA* and *luxB* genes encode α and β subunits of the bacterial luciferase, *luxC*, *luxD* and *luxE* encode enzymes involved in the synthesis of aldehyde substrate utilized in the light emission reaction and *luxG* codes for flavin reductase, which participates in flavin mononucleotide turnover [5], [6].

Until now, expression of various luciferases in plants required exogenous application of luciferins – frequently toxic and high-cost compounds – to achieve only temporary and relatively low light emission levels from live plant tissues [7]. We embarked on an examination to see whether a complete functional bacterial luciferase pathway can be reconstituted in a transplastomic plant to produce both the luciferase and luciferins. Among different independently-evolved luminescent enzymatic apparati, the bacterial light emission system is best suitable for creation of autonomously luminescent plants due to cyanobacterial evolutionary origins of plant plastids [8], [9]. This evolutionary similarity underlies the ability of both plants and bacteria to manufacture riboflavin, from which flavin mononucleotide is produced. The fatty acid biosynthesis pathway, from which the aldehyde substrate is derived, is supported by the same type II fatty acid synthase (FAS II) in both plants and bacteria [10], [11] in contrast to animals and fungi, where fatty acids biosynthesis is mediated by type I fatty acid synthase (FAS I) [12]. Also, plastidal gene expression machinery allows coordinated expression of multitransgene operons [13], [14] and lacks nuclear transgene silencing mechanisms [15], [16], which would be detrimental for expression of a complex pathway involving multiple genes. However, while on the one hand plant chloroplasts share evolutionary origins with prokaryotes, these organelles are not bacteria, and fundamental biological differences between the two exists. For instance, many of the chloroplast encoded open reading frames do not have Shine-Dalgarno sequences, required for bacterial translational initiation, and chloroplast initiation codons are not limited to AUG or GUG as in free living eubacteria [17]. Other differences include coordination of an operon-encoded gene expression, intricacies of transcriptional regulation, posttranscriptional RNA processing, protein folding and quality controls machinery, and other characteristics [18], [19]. It is, therefore, remarkable that higher plants are capable to fully and correctly functionally reproduce a complex enzymatic pathway from an evolutionary unrelated marine organism. While co-expression of a limited number of transgenes in chloroplasts have been investigated [14], a complete and functional foreign biochemical multienzyme pathways have not been reconstructed in plastids. Here, we generated the first truly autonomously luminescent (autoluminescent) transplastomic plants, containing a fully functional bacterial luciferase pathway, which emits visible light detectable by the naked eye.

## Results

### Generation of Autoluminescent *Nicotiana tabacum* Plants

We generated two independent lines of *Nicotiana tabacum* transplastomic plants, carrying the bacterial *lux* operon from *Photobacterium leiognathi*. In one line, the *lux* operon was integrated into the *rps12/TrnV* locus of the chloroplast genome, whereas, in the other line, it was integrated into a more transcriptionally active *TrnI/TrnA* locus [20], [21]. To produce these plants, the *lux* operon, containing the *luxCDABEG* genes was cloned under the control of the tobacco plastidal *Prrn* promoter into the plastid transformation vector pCAS3 (see Methods), which carries a spectinomycin resistance selection marker *aadA*, resulting in pCAS3-aadA-LUX vector. Homologous recombination sites for integration into *rps12/TrnV* or *TrnI/TrnA* tobacco plastid genome loci were inserted to flank the *aadA-lux* expression cassette, resulting in pCA3-LUX-rps12/TrnV or pCA3-LUX-TrnI/TrnA vectors, respectively, and transplastomic plants were generated using standard microbombardment methods and selection on spectinomycin-supplemented media [22], [23].

**Figure 1:**
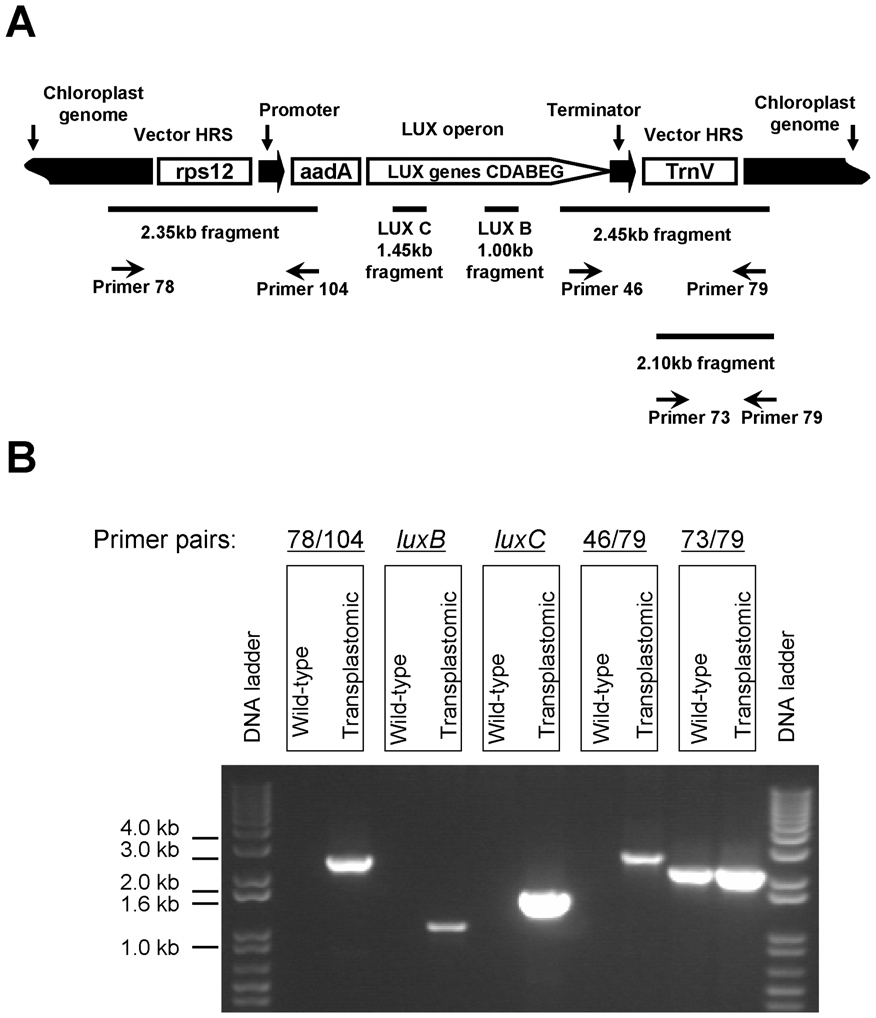
PCR analyses to confirm plastid transformation with vector pCAS3-LUX-rps12/TrnV. (A) Location of PCR primers on the vector and predicted size of junction PCR fragments. Shown are also: the *rps12* and *trnV* plastid genes; *aadA*, the spectinomycin resistance gene; the *lux* genes. The drawing is not to scale. (B) Junction PCR fragments obtained by the primers in Fig. 1A using total cellular DNA of a transplastomic plant line as template.

**Figure 2:**
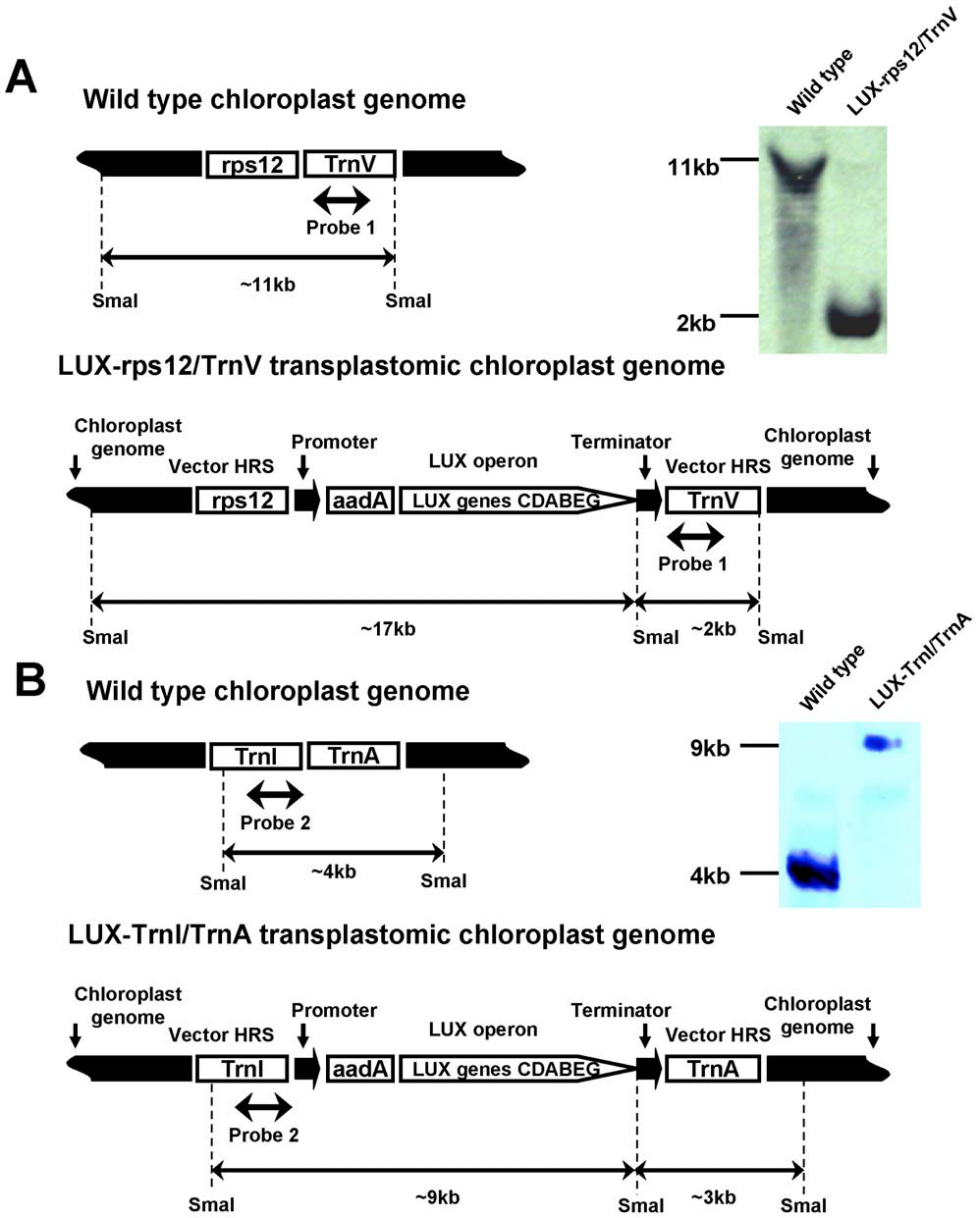
DNA gel blot analysis confirms plastid transformation with vectors pCAS3-LUX-rps12/TrnV (A) and pCAS3-LUX-trnI/trnA (B). Total cellular DNA isolated from transplastomic leaves was digested with the *Sma*I restriction endonuclease and probed with a fragment of the vector plastid-targeting region.

**Figure 3:**
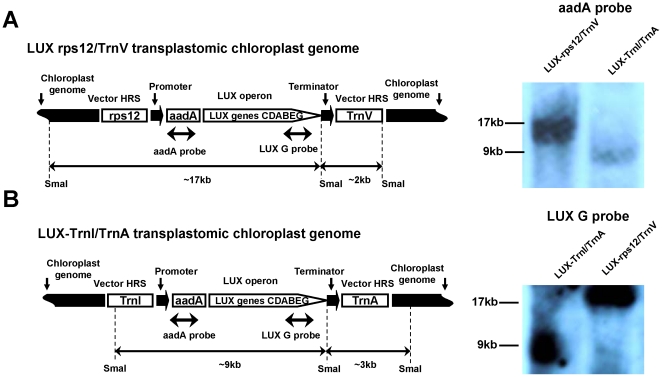
DNA gel blot analysis confirms integration of *aadA* and the *lux* operon in the LUX-rps12/TrnV (A) LUX-trnI/trnA (B) plastid genomes. Total cellular DNA isolated from transplastomic leaves was digested with the *Sma*I restriction endonuclease and probed with a fragment of the vector plastid-targeting region. Shown are also: the *rps12* and *trnV* plastid genes; the *trnI* and *trnA* plastid genes; *aadA*, the selective spectinomycin resistance gene; the *lux* genes. The drawing is not to scale.

Once initial shoots had appeared, we used junction PCR to differentiate true transplastomic plants from small ribosomal RNA (*rrn16*) spontaneous mutants, which are also spectinomycin-resistant [24], [22]. In this approach, one of the PCR primers is located within the expression cassette and the other is positioned within the chloroplast genome, outside of vector sequences, leading to amplification of genome-integrated transgene junction. Fig. 1A illustrates the PCR products predicted to arise from transplastomic plants generated using pCA3-LUX-rps12/TrnV, whereas Fig. 1B demonstrates the results of this junction PCR analysis. The 2.35-kb fragment amplified using primers 78 and 104 and the 2.45-kb fragment amplified using primers 79 and 46 are diagnostic of integration of the entire expression cassette integration into the *rps12/TrnV* locus (Fig. 1B). Next, we amplified *luxB* and *luxC* to further confirm the presence of the *lux* operon within the transplastomic genome (Fig. 1B). The relatively small (∼155 kb) plastid genome in tobacco is present in thousands of copies per cell [24]. Integration of *aadA* and the *lux* operon genes in the LUX-rps12/TrnV genome, and the absence of non-transformed wild-type ptDNA copies has been confirmed by DNA gel blot analyses using plastid targeting sequences, *aadA* and *lux* gene probes (Figs. 2 and 3). Transplastomic plants transformed with the pCAS3-LUX-TrnI/TrnA plasmid were identified in a similar fashion, using junction PCR primers specific for *TrnI* and *TrnA*, as well as for *luxB* and *luxC*. Integration of *aadA* and the *lux* operon genes in the LUX-TrnI/TrnA genome, and the absence of non-transformed wild-type ptDNA copies has been confirmed as described for the LUX-rps12/TrnV plants (Figs. 2 and 3). None of the transplastomic plant lines exhibited detectable phenotypic changes in their development or morphogenesis.

### Characterization of Light Emission Properties of the Autoluminescent Plants

**Figure 4:**
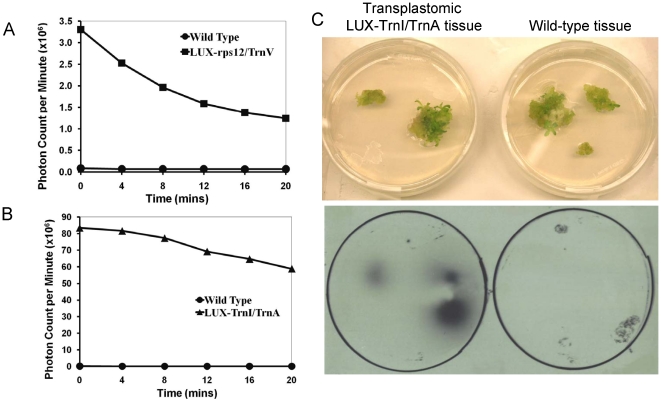
Quantification and autoradigraphic detection of autoluminescence in transplastomic plants. (A, B) Scintillation spectroscopy of transplastomic LUX-rps12/TrnV and LUX-TrnI/TrnA tissues (150 mg), respectively, in a Beckman LS 6500 multi-purpose scintillation counter. (C) Photographs (top panel) and autoradiographs of the LUX-TrnI/TrnA plants (bottom panel).

Next, we examined the luminescence properties of the LUX-rps12/TrnV and LUX-TrnI/TrnA transplastomic plants. For quantification of light emission, emerging transplastomic or wild-type shoots were placed in scintillation counter vials, incubated in the dark for 5–10 min to eliminate chlorophyll autofluorescence, and photon count was recorded for 20 min. Fig. 4 shows that the transplastomic tissues emitted large quantities of photons of visible light, with LUX-rps12/TrnV and LUX-TrnI/TrnA initially emitting around 3.3×10^6^ and 82.0×10^6^ photons/min (panels A, B, respectively), while background noise was measured using wild type tissue and recorded at only 60–70×10^3^ photons/min. The LUX-TrnI/TrnA plants emitted approx. 25 times more photons from the same amount of tissue than the LUX-rps12/TrnV plants (Fig. 4A, B), presumably due to the higher read-through transcriptional activity of the *TrnI/TrnA* locus. The decline in the luminescence during the scintillation readings (Fig. 4A, B) most likely resulted from depletion of oxygen in the tightly closed scintillation vials, which is utilized in the light emission reaction.

**Figure 5:**
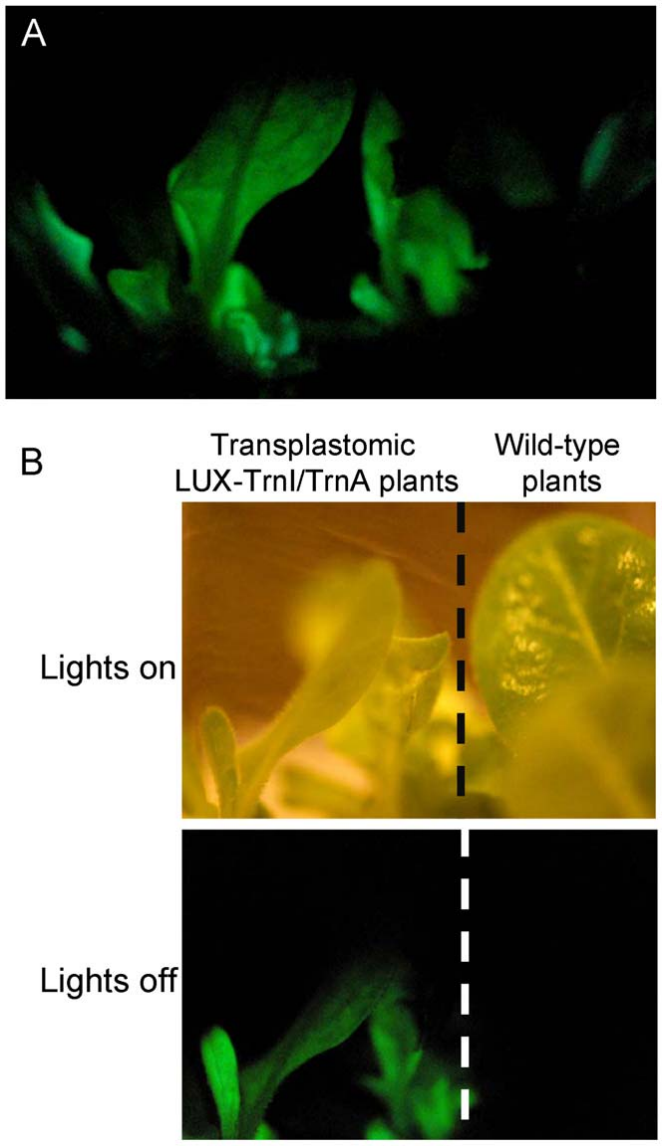
Visual detection of autoluminescence in LUX-TrnI/TrnA plants. (A) Photograph taken in the dark with a hand-held consumer camera (Nikon D200; AF-S Micro Nikkor 105.0 mm 1∶2.8 G ED lens; exposures 5 min at f/4.5, 105mm focal length, ISO 3200). (B) Photographs of transplastomic and wild-type plants taken with lights on or off.

Overnight autoradiography of the LUX-TrnI/TrnA shoots produced a defined and focused image spots around the transplastomic tissue, whereas no such light emission was detected with the wild-type tissue (Fig. 4C). Finally, it was important to demonstrate that the transplastomic plants in fact emit light visible with the naked eye. Indeed, when the fully grown, homoplastomic LUX-TrnI/TrnA plants were placed in a dark room, their glow was clearly seen after about 5–10 min of eye adjustment to darkness. Fig. 5 illustrates the images of these plants as recorded in the dark and in the light, using a standard hand-held consumer camera. No such glow was detected in the wild-type plants.

## Discussion

In this work, we produced the first example of autoluminescent plants, with the glow clearly visible to the human eye. The basic biochemical machinery required by the *lux* operon-encoded enzymatic pathway for light emission is very similar across all plant kingdom, making our approach applicable to essentially any plant species. The plant autoluminescence might be further modified in regard to its intensity through the use of different promoters, modification of luciferase substrate levels in the cell, or enhancement of the luciferase catalytic activity via directed evolution [25]. The color of the emitted light can also be modified via luciferase mutagenesis [26], and glow in specific plant organs can be achieved through the use of chloroplast-targeted nuclear-encoded transcription factors expressed from tissue-specific promoters. Thus, our findings not only enhance our understanding of the fundamental biological processes and expression of complex and fully functional multienzymatic pathways in plant plastids, but also are useful for floriculture industry, particularly since plastid DNA is maternally inherited in most flowering plant species [27], substantially reducing the risks of transgene escape into the environment.

## Materials and Methods

### Chloroplast transformation vectors

Chloroplast transformation vectors of the pCAS series were constructed using the backbone of the pSAT4-MCS vector (GenBank accession number DQ005466.1; [28]). The *CaMV 35S* promoter of pSAT4-MCS was replaced by a chloroplast *Prrn* promoter, cloned as a AgeI/NcoI PCR fragment amplified using forward 5′-TCACCGGTCGCCGTCGTTCAATGAGAATGG-3′ and reverse 5′-GAGCGAACTCCGGGCGAATATCCATGGTT-3′ primers and *Nicotiana tabacum* plastid genomic DNA as a template, resulting in pCAS3 vector. Then, a spectinomycin resistance gene *aadA* fused to an *rbcL* leader sequence was cloned into pCAS3 as a BglII/NcoI PCR fragment amplified using forward 5′-AACCATGGAGTTGTAGGGAGGGATTTATGGGGGAAGCGGTGATCGCC-3′ and reverse 5′-TGGAGATCTTTATTTGCCGACTACCTTGGTGATC-3′ primers and pPZP-RCS2 [28] as a template, producing pCAS3-aadA. The *lux* operon from *Photobacterium leiognathi* (GenBank accession number M63594), comprising *luxCDABEG*, was cloned as an EcoRI PCR fragment amplified using forward 5′-ACAGAATTCCCAAAGGAGATTACATGATTAAG-3′ and reverse 5′- TTGGAATTCTTACGTATAGCTAAATGCATCAG-3′ primers and *Photobacterium leiognathi* genomic DNA as a template into the same site of pCAS3-aadA, resulting in pCAS3-aadA-LUX.

To allow integration into the *rps12/TrnV* or *TrnI/TrnA* loci, the corresponding homologous recombination (HR) sequences were amplified from the *Nicotiana tabacum* plastid genomic DNA and inserted to flank the *lux* operon expression cassette in pCAS3-aadA-LUX. For pCA3-LUX-rps12/TrnV, the *rps12* HR sequence was first cloned as an AgeI PCR fragment amplified using forward 5′-AGTTAGAACCGGTGAAGTGCTTCGAATCATTGCTATTTG-3′ and reverse 5′-CGATCTAACCGGTTTATCAACTGCCCCTATCGGAAATAGG-3′ primers. The *TrnV* HR sequence was then cloned into the resulting vector as an NcoI PCR fragment amplified using forward 5′-ATAATGCGGCCGCCAATTGAATCCGATTTTGACCATTATTTTC-3′ and reverse 5′-ATTATGCGGCCGCGTGAAGCAGTGTCAAACCAAAATACC-3′ primers. For pCA3-LUX-TrnI/TrnA, the *TrnI* HR was first cloned as an AgeI PCR fragment amplified using forward 5′-AGTTAGAACCGGTCTTCGGGAACGCGGACACAGGTGG-3′ and reverse 5′-CGATCTAACCGGTAGATGCTTCTTCTATTCTTTTCCCTG-3′primers. The *TrnA* HR sequence was then cloned into the resulting vector as a NotI PCR fragment amplified using forward 5′-CTATTATGCGGCCGCACTACTTCATGCATGCTCCACTTGG-3′ and reverse 5′- GAATGATGCGGCCGCCCTATGAAGACTCGCTTTCGCTACG-3′ primers. All constructs were verified by DNA sequencing.

### Transplastomic plants

Transplastomic *Nicotiana tabacum* (cv. Petit Havana) plants were produced by standard chloroplast transformation protocols [22], [23]. Homoplastomy was confirmed by the Southern blot analysis.

### Junction PCR primers

Transplastomic LUX-rps12/TrnV plants were identified using primers 78 (5′-TTGAGTATCCGTTTCCCTCC-3′) and 104 (5′-CCAGCAAATCAATATCACTGTGTGG-3′), which produced a 2.35-kb fragment, and primers 46 (5′-CAGATTTATCTGACTTTGATATCTATG-3′) and 79 (5′-AAGCTCATGAGCTTGGTCTTAC-3′), which produced a 2.45-kb fragment. Transplastomic LUX-TrnI/TrnA plants were identified using primers (5′-CGTTCGCAAGAATGAAACTCAAAGG-3′) and (5′-CAACATCACTTTGGGTGATGATAGG-3′) producing approx. 2.8kb fragment and specific for *TrnI* junction, and primers (5′-CAGATTTATCTGACTTTGATATCTATG-3′) and (5′-CGCTGATTCTTCAACATCAGTCG-3′) producing approx. 2.2kb fragment and specific for the *TrnA* junction. The *luxB* and *luxC* genes were amplified using primer pairs 5′-ATGAATTTCGGGTTATTTTTCC-3′/5′-TTATTTAATAAGGTTATCTTTG-3′ and 5′-ATGATTAAGAAGATCCCAATGA-3′/5′-CTACGGTACAAATACGAGGAAC-3′, respectively. Primers 73 (5′-AATTGAATCCGATTTTGACCATTATTTTC-3′) and 79 (5′-AAGCTCATGAGCTTGGTCTTAC-3′) amplified an intact region of the chloroplast genome for positive controls.
